# Consideration of the role of protein quality in determining dietary protein recommendations

**DOI:** 10.3389/fnut.2024.1389664

**Published:** 2024-11-13

**Authors:** Robert R. Wolfe, David D. Church, Arny A. Ferrando, Paul J. Moughan

**Affiliations:** ^1^Department of Geriatrics, University of Arkansas for Medical Sciences, Little Rock, AR, United States; ^2^Riddet Institute, Massey University, Palmerston North, New Zealand

**Keywords:** protein quality, essential amino acid, dietary requirements, dietary protein, protein scoring

## Abstract

The quality of a dietary protein refers to its ability to provide the EAAs necessary to meet dietary requirements. There are 9 dietary amino acids that cannot be metabolically produced in the body and therefore must be consumed as part of the diet to avoid adverse metabolic consequences. These *essential amino acids* (EAAs) serve a variety of roles in the body. The amount and profile of the dietary EAAs relative to the individual EAA requirements and the digestibility of the dietary protein are the key factors that determine its quality. Currently the Digestible Indispensable Amino Acid Score (DIAAS) is the best available approach to quantifying protein quality. The most prominent metabolic role of dietary EAAs is to stimulate protein synthesis by serving as signals to activate molecular mechanisms responsible for the initiation of protein synthesis and, most importantly, to provide the necessary precursors for the synthesis of complete proteins. Current dietary recommendations generally do not consider protein quality. Accounting for protein quality in dietary patterns can be accomplished while staying within established ranges for dietary protein consumption. Poor protein quality can be compensated for to some extent by eating more low-quality protein, but to be effective (“complementary”) the limiting EAA must differ between the low-quality protein and the base diet to which it is being supplemented. Adding a high-quality protein to a dietary pattern based on low-quality protein is more effective in meeting EAA goals than increasing the amount of low-quality protein, even if the low-quality proteins are complementary. Further, reliance entirely on low-quality protein food sources, particularly in circumstances that may benefit from a level of dietary EAAs greater than minimal requirements, is likely to include excessive caloric consumption. While protein consumption in high-income nations is generally perceived to be adequate or even excessive, assessment of dietary patterns indicates that a significant percentage of individuals may fall short of meeting optimal levels of EAA consumption, especially in circumstances such as aging in which the optimal EAA consumption is greater than basal values for healthy young individuals. The case is made that protein quality is an important consideration in meeting EAA requirements.

## Introduction

Dietary protein has been recognized for more than 100 years as vital for growth, health, and even survival (1). Amino acids are the building blocks of dietary protein, and it is the amino acids absorbed from digested dietary protein that serve the various metabolic roles. Dietary amino acids serve as precursors for the synthesis of neurotransmitters, nucleotides, and a variety of other important products. Dietary amino acids also support multiple aspects of immune function, and influence satiety. Most prominently, dietary amino acids serve as precursors for the synthesis of new proteins in the body. There are thousands of different proteins in the body, all with specific functions. Proteins comprise about two-thirds of the mass of the body that is not water. Each protein is distinguished by the unique amount and profile of amino acids of which it is composed. All proteins in the body are in a constant state of turnover, meaning continuous breakdown and synthesis (2). Protein turnover enables a replenishing of older, less functional proteins with new, better-functioning proteins (3). Most adults are in a steady state in which the synthesis of proteins over the course of the day balances breakdown.

Protein turnover proceeds continuously throughout the day and night, regardless of whether amino acids from dietary protein are being absorbed. The amount of time throughout the day that dietary amino acids are being absorbed varies according to patterns of consumption. Eating patterns vary in different cultures. In the United States, it is common to eat discrete meals (usually three) per day containing dietary protein, but most protein is often consumed in the evening meal. The consumption of discrete meals results in periods of 3–6 h each throughout the day during which amino acids are absorbed (post-prandial state), depending on the composition of the meal. Regardless of the pattern of consumption of dietary protein there are periods when dietary amino acids are not being absorbed (post-absorptive state). The post-absorptive state is characterized by a net breakdown of body proteins due to the rate of protein breakdown exceeding the rate of protein synthesis. Although the amino acids released by protein breakdown can serve as precursors for the synthesis of new proteins, the availability of certain amino acids from protein breakdown is insufficient to allow protein synthesis to balance the rate of breakdown in the post-absorptive state because of the irreversible oxidation of those amino acids and the inability of the body to replace them metabolically. Also, amino acids are lost directly from the body via routes such as the gastrointestinal tract and skin. The amino acid components of body protein that cannot be synthesized in the body are called the dietary *essential amino acids* (EAAs). The necessity of including EAAs in the diet has been recognized for close to 100 years (4). The EAAs for human nutrition are histidine, leucine, lysine, isoleucine, methionine, phenylalanine, threonine, tryptophan, and valine. There are an additional 11 dietary dispensable amino acids that are also components of body proteins but can be produced in the body. The extent of oxidation of each EAA released in the process of protein breakdown largely defines its dietary requirement. Consumption of at least that amount of each EAA is necessary to maintain protein balance over the course of the day.

The post-absorptive state generally lasts for a matter of hours, but it is possible for humans to survive for a month or more without dietary protein consumption (5). Protein turnover occurs in all tissues and organs in the body and sustained negative protein balance in certain tissues and organs, such a skin, heart, brain, etc. is not compatible with life. In this circumstance skeletal muscle serves as a “reservoir” of amino acids for the tissues and organs with a high priority to maintain protein balance. The net breakdown of muscle protein and release of amino acids into plasma in the absence of dietary protein intake enables sufficient availability of EAAs to maintain protein balance in the other tissues and organs in the body.

While consumption of dietary protein promotes protein synthesis throughout the body, stimulation of *muscle* protein synthesis in the post-prandial state to replenish protein lost in the post-absorptive state is a primary metabolic role of dietary protein. Skeletal muscle protein metabolism is not only central to maintaining protein homeostasis throughout the body, but muscle serves a variety of other roles. The importance of maintaining muscle mass and function in relation to physical activity is well-known (6). Less well appreciated, skeletal muscle protein turnover plays an important role in maintaining energy balance, as both muscle protein synthesis and breakdown require energy in the form of ATP (7). The difficulty in maintaining weight loss after caloric restriction weight loss is related in part to the extent of loss of muscle mass (8). Maintaining the metabolic function of muscle is central to avoiding metabolic syndrome and type 2 diabetes, since muscle is the primary site of glucose clearance from plasma (7). Muscle contraction puts torque on bone that is essential for bone strength (9). These multiple and varied roles of skeletal muscle are important for all individuals, and especially for vulnerable populations such as the elderly, and must be supported by adequate EAA consumption.

The quality of a dietary protein can be described as its ability to provide the EAAs necessary to maintain protein balance in the body by stimulating protein synthesis. Evaluation of the importance of protein quality therefore requires consideration of the role of EAAs in stimulating protein synthesis (10), and the factors that determine the effectiveness of the dietary protein in delivering the necessary EAAs to the tissues and organs of the body. These factors include the amount and profile of EAAs in a dietary protein relative to nutritional requirements, and the digestibility of the protein. Fundamental issues related to the importance of protein quality include the scoring of protein quality, the relation between the true ileal digestibility of dietary EAAs and the stimulation of protein synthesis, the mechanism of stimulation of protein synthesis by EAAs, the accuracy of EAA requirements that are targeted in assessing protein quality, whether consideration of protein quality can be incorporated into diet planning while staying within established nutritional recommended ranges for dietary protein consumption, the effect of physiological and metabolic circumstances on optimal EAA consumption the significance of non-protein components of protein food sources, if poor protein quality can reasonably be compensated for by eating more protein, and the relevance of the quality of dietary protein in high-income nations. We will briefly discuss these issues in relation to the Digestible Indispensable Amino Acid Score (DIAAS) to quantify protein quality.

### Scoring protein quality: a case for DIAAS

A variety of approaches have been used to describe the quality of a dietary protein. Some approaches have been based entirely on digestibility. These include oro-ileal true amino acid digestibility, total (fecal) crude protein digestibility and a dual isotope tracer method that compares circulating amino acids from an intrinsically labeled test protein with a reference protein labeled differently with known digestibility (11). Biological value (BV) is based on measures of nitrogen digestibility and urinary nitrogen excretion, whereas net protein utilization (NPU) is based on nitrogen intake and urinary nitrogen excretion. The Protein Efficiency Ratio (PER) reflects the physiological response to the dietary protein and is based on the ratio of weight gain to protein consumed by the test group as compared to the control (most commonly casein) over time (12). The first efforts to consider EAAs as individual nutrients and to assign a numerical value to the quality of a dietary protein involved chemical scores such as the Protein Digestibility Corrected Amino Acid Score (PDCAAS). PDCAAS is based on the fecal digestibility of crude protein and the content and profile of EAAs (11). The Digestible indispensable Amino Acid Score (DIAAS) was published in 2013 as a more accurate estimation of the factors comprising PDCAAS (13). The underlying concept of protein quality as quantified by both PDCAAS and DIAAS is that the amino acids in a dietary protein must be digested and absorbed to have metabolic value, and that the amount and profile of the absorbed EAAs should be in line with the dietary requirements for each EAA. The improved accuracy of DIAAS as compared to PDCAAS derives from the use of true ileal digestibility of each essential amino acid (EAA) in the dietary protein rather than the fecal digestibility of crude protein, and DIAAS (but not PDCAAS) is not truncated in the case of high-quality proteins. DIAAS also specifically accounts for the availability of lysine in processed foods. While DIAAS most conventionally applies to single dietary proteins, the DIAAS of a complete diet can also be calculated (13). Thus, DIAAS is the most accurate method currently available to provide a basis for dietary recommendations for protein consumption to account for quantitative differences in dietary protein quality. The validity of DIAAS for this purpose has two principal aspects: the use of true ileal amino acid digestibility and the role of EAAs in controlling protein synthesis in the body. We have previously analyzed the validity of the factors comprising DIAAS in depth (14). One possible shortcoming of DIAAS is that the value is expressed in terms of the percent of dietary EAA requirements met when the EAR for protein is consumed. Using the EAR value is useful on a population basis, but it may be more appropriate to express the percent of requirements met when the RDA for protein is consumed when determining dietary recommendations on an individual basis.

### Use of true ileal amino acid digestibility (TID) in quantifying protein quality

It is self-evident that for a dietary protein to have metabolic value it must be digested and the amino acids absorbed. Thus, there is little argument that an accurate scoring of protein quality should take account of digestibility. There are two basic approaches to determining protein digestibility directly: fecal or ileal digestibility. Digestibility determined at the ileal level is fundamentally superior to determining digestibility at the fecal level since there is little absorption of amino acids in the large intestine and there is an abundance of microflora that digests and utilizes undigested protein, peptides or amino acids exiting the small intestine (15, 16). In addition, amino acids can also be synthesized and microbial degradation products absorbed in the large intestine (17). The catabolism and synthesis of amino acids by the microflora in the large intestine confounds fecal measurements of protein or amino acid digestibility and will usually result in the over-estimation of the true digestibility of the EAAs in the test protein. Further, the amino acid composition of fecal protein bears no necessary resemblance to the undigested dietary protein leaving the ileum. Accounting for digestibility at the end of the small intestine (ileal digestibility), as is done with calculation of DIAAS, overcomes the problems of interpreting fecal digestibility data.

Use of true ileal amino acid digestibility (TID) in quantifying protein quality is important because TID can vary across amino acids, even within the same protein source. For example, TID of dietary proteins in India was found to differ by more than 20% across the dietary EAAs for many foods and food ingredients examined (18). Even for highly digestible protein sources the range in true ileal amino acid digestibility within a protein source can be significant (18). TID generally varies more in plant-based dietary proteins than animal proteins. For example, TID of EAAs in beef sirloin ranges from 98–100%, while the corresponding measurements in boiled potato protein range from 56% (tryptophan) to 83% (lysine) (19). Failure to take account of true ileal digestibility in this example would result in not only an overestimation of the quality of potato protein but would change the limiting amino acid from histidine to lysine. This is not to imply that all plant proteins have low and variable digestibility. For example, amino acid digestibility is relatively high for soy isolate, but in general animal proteins have higher and less variable digestibility. The main concerns with ileal measurement of amino acid digestibility include how well digesta samples reflect the total digesta, if the contribution of the non-dietary EAAs derived from digestion of digestive enzymes and other intestinal proteins has been accurately accounted for, and whether any effects that small intestinal bacteria may have on digestibility is considered. The primary factor limiting the use of TID in scoring protein quality is that values have not been determined in some dietary proteins A major effort to determine TID in a wide range of dietary proteins is under way and completion of that work will enable a broader application of TID in scoring protein quality.

### EAAs and protein synthesis

In addition to TID, accurate scoring of protein quality must account for the amount and profile of the EAAs in a test protein relative to the corresponding values in the reference protein, and the accuracy of EAA requirements on which the amino acid scoring pattern of the reference protein is based (14). The mechanisms responsible for how EAAs regulate protein synthesis are thus central to understanding the basis for DIAAS. Further, accounting for how EAAs regulate protein synthesis is important in determining the adequacy of protein consumption in a variety of physiological states.

**Figure 1:**
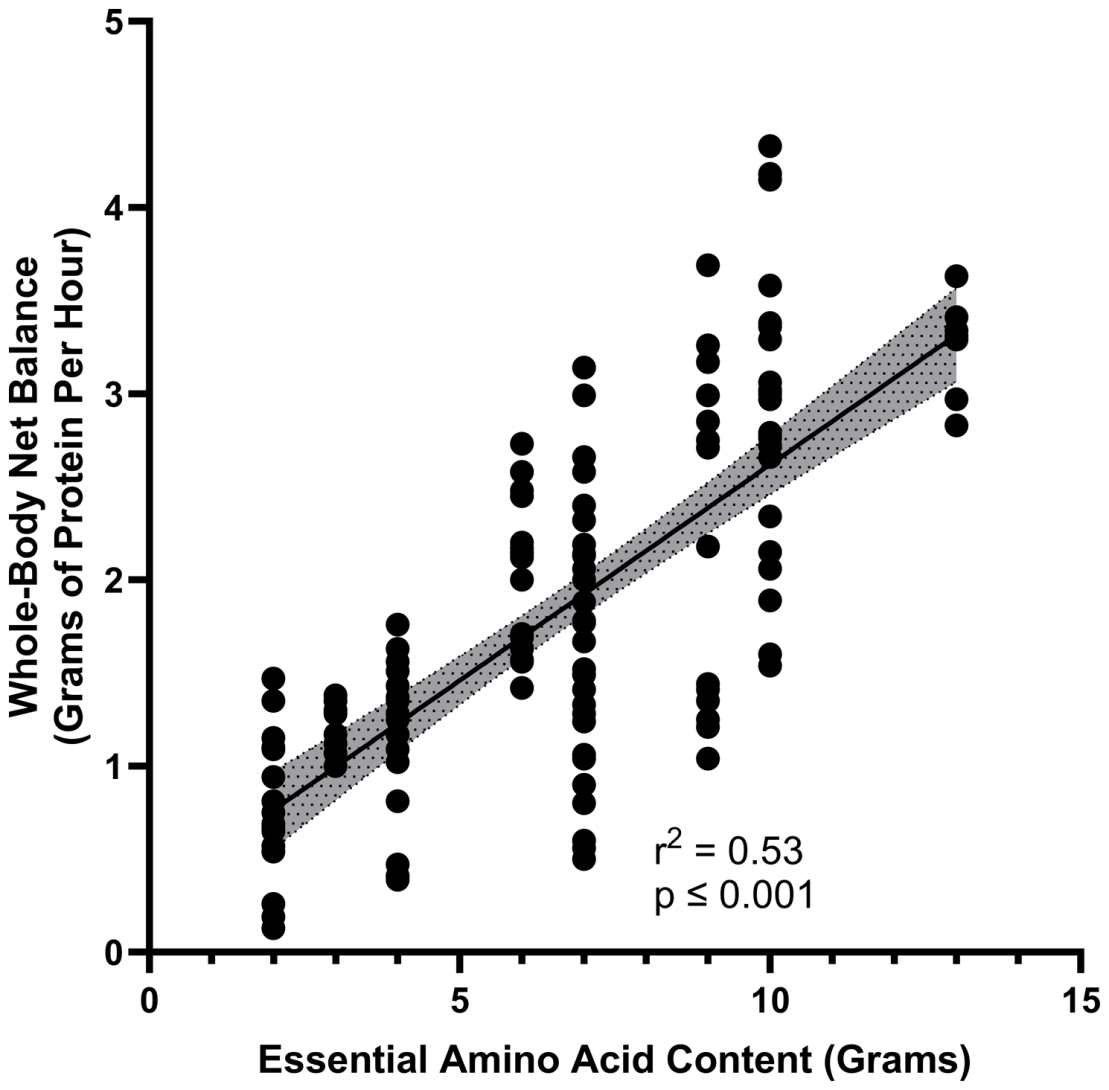
Relationship between increase in essential amino acid content of a protein source and the gain in whole-body protein balance (represented as grams per hour) (14, 15).

Dietary EAAs are primarily responsible for the stimulation of protein synthesis in the post-prandial state. Consumption of a relatively small dose of only EAAs in the profile of beef protein stimulates muscle protein synthesis (MPS) as much as a mixture of the same amount of EAAs plus additional dispensable amino acids (DAAs) that can be produced in the body (10). When only EAAs are consumed the DAAs that are also required for the synthesis of new proteins can be derived from reutilization of endogenous DAAs released by protein breakdown or synthesized in the body, often from simple nitrogenous precursors. In contrast to the stimulatory effect of EAAs on protein synthesis, ingestion of a mixture of DAAs in the profile found in whey protein failed to stimulate MPS (20). Further, the magnitude of increase in whole-body protein synthesis appears to be directly related to the amount of EAAs in a dietary protein, provided that high-quality proteins with high digestibility are considered (21, 22) (Figure 1). Dietary proteins with low digestibility would not yield the same relation between the amount consumed and the stimulation of protein synthesis.

While the EAAs are primarily responsible for the stimulation of protein synthesis, the dietary DAAs may also play a role. The importance of dietary DAAs in maintaining N balance was documented in the early studies of amino acid metabolism. The efficiency of utilization of the EAAs as assessed by N-balance was shown to be enhanced by the amount of DAAs given concurrently (23). The exact amounts of either total or individual dietary DAAs that are necessary to maximize the effectiveness of dietary EAAs have not been determined. Agricultural science literature indicates that the ideal composition of feed for the maximum growth and muscle development of farm animals consists of approximately two-thirds amino N in the form of EAAs (24), but comparable data for humans are not available. Thus, although there is some (uncertain) need for DAA intake, it is most likely that the prevalence of DAAs and other nitrogenous compounds in dietary protein is more than adequate to provide ample DAAs when sufficient protein is ingested to meet EAA requirements. Dietary protein ranges between 30 and 50% EAAs, which means that the contribution of DAAs to amino acid composition of proteins is likely more than adequate to meet requirements if the animal literature can be extrapolated to human diets. Further, normal dietary consumption of DAAs is sufficient to support protein synthesis resulting from ingestion of a relatively small amount of free EAAs (25, 26).

### Mechanisms of stimulation of protein synthesis by EAAs

Measures of protein quality must be consistent with the mechanisms whereby EAAs stimulate protein synthesis. Much of what we know about EAAs and protein synthesis in humans comes from studies of muscle protein synthesis (MPS). The mechanisms whereby EAAs affect protein synthesis in general and MPS specifically fall into two general categories: transcription and translation. The transcription of messenger RNA (mRNA) from DNA results from activation of the relevant genes. Activation of genes is reflected in the number of specific mRNAs in the cell on which the assembly of new proteins occurs. Several studies have used mRNA content of specific proteins as an index of the rate of synthesis of those proteins, but there is generally a poor correlation between mRNA content and MPS (27). Consequently, it is likely that in most circumstances, mRNA content is not rate limiting for MPS.

The translational control of protein synthesis by EAA availability has been recognized since 1958 (28). Translation involves the sequential bonding of amino acids in the order dictated by the mRNA code. Free intracellular amino acids are bound to specific transfer RNAs (tRNAs) inside the cell that have codons of three nucleotides that correspond to the codons on the mRNA for specific amino acids. Charged tRNA molecules sequentially transfer the attached amino acids to the sites on the mRNA dictated by the mRNA code. Translational elongation can only proceed to completion if adequate amounts of all required amino acid precursors are available. A relative deficiency of any EAA will make that EAA limiting. Lack of availability of the limiting EAA will cause the termination of translational elongation of protein synthesis before the process is complete, and the partially synthesized protein being degraded.

Translation of the mRNA is initiated by a complex process which consists of several linked stages that are mediated by eukaryotic initiation factors (eIFs). The mammalian target of rapamycin complex 1 (mTORC1) is a key regulator of the activation of downstream eIFs that are mediators of MPS initiation. Translational initiation of the protein synthetic process can be stimulated by an increased availability of EAAs, and leucine in particular is a potential regulator of mTORC1 (29, 30). The activation of mTORC1 by leucine seems to be especially important in anabolic-resistant states such as aging (31). When older individuals were given a mixture of EAAs in the profile of whey protein the net anabolic response of muscle protein increased only about half of the amount of the response to the same mixture of EAAs in younger individuals (32). Decreased responsiveness of MPS to nutritional stimulation is termed *anabolic resistance*. When comparable older individuals consumed a different mixture of the same amount of EAAs in which leucine comprised approximately 35% of the total mixture, the anabolic response doubled but the enhanced mixture had no greater effect in younger individuals than the profile of EAAs in whey protein (33). The potential role of leucine in triggering the initiation of protein synthesis highlights the importance of considering protein quality in designing dietary plans, particularly in circumstances such as aging (34). The concentration of leucine in plasma must increase approximately 3- fold to activate mTORC1 (35), which translates to consumption of approximately 2.5–3 g of leucine. A relatively large proportion of a dietary protein must be comprised of leucine to achieve that level of intake. Circumstances benefitting from a high leucine intake generally means reliance on animal proteins, which generally contain greater amounts of leucine than plant-based dietary proteins (36), although there are some specific plant proteins that contain relatively high amounts of leucine.

The prevalence of all the EAAs, and perhaps specific EAAs such as leucine, is thus an important aspect of protein quality. In addition, the amount of the limiting EAA in a dietary protein determines the amount of body protein that can be synthesized.

### Use of DIAAS to evaluate protein quality of a meal

DIAAS was developed for the comparison of the quality of dietary proteins, with a particular focus on regulatory issues. For this reason, DIAAS is normalized for the amount of the test protein. As such, DIAAS is not directly relevant to the protein quality of a meal or dietary patern. DIAAS can be calculated for mixtures of proteins as occurs in a meal, but the DIAAS of different proteins in a mixture is not additive because the DIAAS of individual proteins in a meal may be based on different limiting amino acids. Rather, the digestible amounts of each amino acid in a meal are additive. As referred to in FAO Dietary Protein Quality Evaluation in Human Nutrition 13, each EAA in a meal should be treated as an individual nutrient. Thus, the amount of each EAA in the meal, corrected for TID of that amino acid, is compared to the amino acid scoring pattern to determine how well each EAA meets dietary requirements. Whereas the amino acid scoring patterns for DIAAS conventionally match the individual EAA requirements as promulgated by the FAO, alternative scoring patterns to determine the adequacy of each EAA in a meal can be used to better match specific circumstances, such as aging, exercise, etc. Better defining appropriate amino acid scoring patterns for different circumstances should be a high research priority.

### Required vs. flexible protein and EAA consumption

Accounting for protein quality in formulating dietary guidelines could impact current recommendations for protein intake. If a diet is comprised of predominantly low-quality proteins, a level of protein consumption greater than the RDA of protein could potentially be necessary to meet all EAA requirements. It is reasonable to evaluate if it is possible to increase dietary protein consumption above the RDA and stay within recommended dietary guidelines. The Dietary Reference Intakes published by the US Institute of Medicine cites the RDA for protein as 0.8 g high-quality protein /kg/day, and the RDA for carbohydrate as 130 g /day (37). There is no RDA for fat intake, but the adequate intake (AI) of linoleic acid is given as 17 and 12 g/day for men and women, respectively, and the AI for linolenic acid is 1.5 and 1.1 g/day for men and women, respectively (37). For a representative 30-year-old adult man weighing 80 kg these recommendations correspond to 256 kcal/day of protein, 520 kcal/day of carbohydrate, and 166 kcal/day of fat, for a total of 942 Kcal/day. The total energy requirement for such a man is dependent not only on body weight but also height, sex, and activity level. An average daily energy expenditure for the representative man is approximately 3,000 kcal/day, and the corresponding value is approximately 2,500 kcal for a representative woman (37). These recommendations indicate that the required amounts of protein, carbohydrate and fat constitute as little as 30–40% of the total caloric requirement to maintain energy balance. The remaining 60–70% of energy consumption could be considered to be discretionary. While it would be reasonable for part of the discretionary energy consumption to be in the form of dietary carbohydrates and fat, it is equally reasonable that dietary protein consumed at a rate greater than the RDA would comprise at least a component of the discretionary energy intake. Increasing dietary protein intake above the RDA is consistent with the Acceptable Macronutrient Distribution Range (AMDR) also published in the Dietary Reference Intakes (37). The AMDR for protein ranges from 10 to 35% of total energy intake; the RDA for protein accounts for approximately 10% of energy intake. These data indicate that an increase in dietary protein consumption well above the RDA to accommodate a greater need for EAAs can be accomplished while staying within current guidelines for dietary protein consumption.

A potential problem with dietary protein constituting as much as 30–35% of protein is whether all nutrient requirements can be met, particularly with a relatively low caloric content. We performed a modeling exercise in which two single-day menus were created, each consistent with the USDA food group serving recommendations for a (relatively low) 2000-kcal healthy U.S.-style eating pattern (38). We found a diet with 30% of energy derived from protein can be achieved without compromising food group serving intake recommendations for fruits, vegetables, grains, including whole grains, and dairy foods, meeting all nutrient requirements (38). A variety of sources of high-quality protein food sources, including fish, poultry, milk, and cheese in addition to meat were used in the diets. It was necessary to rely on these sources of protein in the meal plans, as the protein density relative to total calories in plant-based protein sources alone is generally low. A meal plan relying on plant-based protein sources alone could only be used in a meal plan targeting a higher caloric intake (38). The necessity of relying on animal proteins in this modeling exercise implies an importance of protein quality in not only providing an optimal level of EAAs, but also meeting all other nutrient requirements. It is possible, and potentially desirable, to increase intake of plant-based proteins, but protein quality needs to be carefully considered. High-quality plant proteins (eg, soy) and animal proteins play an important role in maintaining overall dietary protein quality.

### Physiological circumstances benefitting from increased protein and EAA consumption

The preceding discussion makes clear that current estimates of requirements for dietary EAAs are minimal values and suggest that there is room within traditional nutritional recommendations for a level of protein intake that exceeds the RDA to optimize EAA consumption. It is therefore relevant to evaluate if different physiological circumstances increase the optimal level of protein and EAA consumption significantly above the RDAs. It is of further interest if the optimal profile of EAAs may differ in various physiological circumstances from the profile of the FAO reference protein, which is predicated on basal EAA requirements in young, healthy individuals.

Several studies have demonstrated beneficial effects of dietary protein intake greater than the RDA and that EAAs are primarily responsible for the responses. For example, increased dietary protein improves muscle mass and strength in older individuals (39), and EAAs can be credited with the beneficial response. Supplementation of the normal diet with 11 g twice per day of EAAs in older individuals improved LBM, strength and functional tests (40, 41). When low-function elderly consumed increased dietary protein in the form of whey protein (DIAAS = 0.96) for 16 weeks, muscle strength and function were significantly improved as compared to a control group given only nutritional education (42). Improvements in all aspects of physical function measured were greater when the same amount of EAAs as whey protein were provided, indicating that the EAA component of the whey protein was responsible for the improvements in physical function (42). Similarly, the loss of LBM and muscle strength that occurred with 28 days of bed rest in healthy young subjects (43, 44) as well as 10 days of bed rest in elderly individuals (45) was ameliorated by supplementation with additional dietary protein or EAAs. The results from the bed rest studies are particularly significant because all known factors other than total protein or EAA intake that might potentially affect LBM changes, including activity and other macronutrient intake, were completely controlled. Increased EAA consumption has also been shown to have beneficial effects in a variety of circumstances, including rehabilitation (46–49); stroke (50, 51); peripheral artery disease (52); renal failure (53–57) inflammation (58, 59); critical illness (60); lung cancer (61); cystic fibrosis (62); chronic obstructive pulmonary disease (63–65); wound healing (66); brain injury (67, 68); metabolic syndrome and cardiovascular risk factors (69–71); obesity (8, 72); liver fat (69, 73–75); and diabetes (76–80).

The optimal profile of dietary EAAs may also be affected by different physiological circumstances. For example, selective oxidation of leucine or the branched chain amino acids commonly occurs in stressful conditions, such as serious injury or illness, due to activation of branched chain keto-dehydrogenase (81). Aerobic exercise causes a greater increase in the oxidation of leucine as compared to the other EAAs (82). Further, some conditions involving anabolic resistance, such as aging, may stem in part from decreased activity of mTORC1. In this case a greater proportion of leucine in a dietary protein or a composition of EAAs may serve as a nutraceutical by activating mTORC1 (29, 30).

While the many studies that have reported beneficial effects of increasing EAA consumption provide a strong rationale for quantifying protein quality on the basis of the EAA profile and amount, it should be recognized that many of the above-mentioned studies used free EAA compositions to raise EAA consumption. It is unclear if a reasonable amount of even high-quality dietary protein alone can elicit the same metabolic and physiological responses. Plasma EAA concentrations increase more rapidly and to higher levels when free EAA compositions are consumed than when the same EAA are components of dietary protein (22). Further, free EAA mixtures often have little or no associated DAAs or non-protein components that may elicit different physiological responses than dietary protein food sources.

In contrast to the many studies demonstrating beneficial effects of increased protein and EAA consumption, there has never been a study to our knowledge in which the RDA for protein or EAA consumption was compared with a higher level of protein intake and the lower level of protein consumption was found to be superior.

An ideal approach to scoring protein quality would account for known effects of specific physiological circumstances on optimal EAA consumption at a group level, and even at an individual level (i.e., “*personalized nutrition*”). A recent publication describes such an approach for any situation in which the optimal amount and profile of EAAs is known (83). DIAAS can also account for altered demand for EAAs in specific circumstances by using a scoring pattern reflecting the optimal amount and profile of EAA consumption for that circumstance rather than the FAO scoring pattern based on the RDAs for the individual EAAs. However, the value of any protein quality scoring is dependent on the accuracy of the target for EAA consumption, and more data in this regard may be necessary for the successful implementation of personalized nutrition.

### Current protein nutrition guidelines

Recommendations for dietary protein intake have been expressed in terms of grams of protein or nitrogen (N) per day for more than 100 years. While occasionally the proviso that recommendations apply to “good quality” protein has been included (e.g., DRIs), in general protein recommendations have not directly specified the source or quality of dietary proteins.

Evaluation of the relevance of protein quality to nutritional guidance is timely. The Dietary Guidelines for Americans (DGAs) published by the United States Department of Agriculture (USDA) have made a pronounced shift over the past 40 years away from animal protein toward plant-based protein food sources. Such a shift may have a significant impact on the overall protein quality of the diet, as animal proteins generally are higher quality (as reflected by DIAAS) than are plant-based proteins (14). Although only about 5% of the US population classifies themselves as vegetarian and about 2% classify themselves as vegan (84), there has been a progressive shift away from consumption of animal-based protein food sources in individuals who do not consider themselves to be vegetarian. For example, between 1970 and 2005 there was a 17% drop in consumption of red meat and of eggs in the U.S. (85), and the downward trend in red meat consumption has continued, in part due to perceived concerns about health and growing publicity regarding the environmental impact of the beef industry in particular (86). However, calls for reduced consumption of animal-based protein food sources have not taken account of the potential physiological implications of a significant reduction in the overall protein quality of the diet.

### “Ounce equivalents” of dietary protein

The DGA’s aim to create recommended dietary patterns that meet or exceed RDAs for both micro- and macronutrients. Levels of protein intake are not the primary focus of the DGAs, perhaps in part because the RDA for dietary protein can be met with almost any western diet that maintains caloric balance. However, the RDA expresses the minimal amount of dietary protein consumption necessary to avoid deficiencies in young, healthy individuals, and, as discussed above, there are many circumstances in which the optimal amount of dietary protein may be greater than the RDA. Further, the DGAs do not currently address the issue of protein quality. DIAAS indicates that animal proteins can more readily provide the daily requirement of EAAs than plant proteins (14).

MyPlate is designed to simplify for the public the key elements of the DGAs (87). MyPlate recommends meeting protein needs by eating a variety of “ounce equivalents” of protein food sources. The DGAs state that 1 ounce (28 g) of meat is equivalent to 1 cooked egg, ¼ cup (70 g) of red kidney beans, 1 tablespoon (15 g) of peanut butter, 2 ounces (56 g) of tofu and 0.5 ounces (14 g) of mixed nuts. The labeling of these disparate protein food sources as “equivalents” implies an equal metabolic benefit should be obtained from each of the “ounce equivalents” of protein food sources, although neither the DIAAS nor the amount of EAAs provided are in fact equivalent. To determine if the different protein food sources provide the same anabolic stimulus, stable isotope tracer methodology was used to quantify the response to ingestion of each of the “ounce equivalents” (88). The changes from baseline following consumption of one of seven different protein food sources were compared to the baseline value for that individual. Consumption of ounce equivalents of animal-based protein food sources (beef sirloin, pork loin, eggs) resulted in a greater gain in whole-body net protein balance above baseline than the ounce equivalents of plant-based protein food sources (tofu, kidney beans, peanut butter, mixed nuts; *p* < 0.01). Most importantly, the magnitude of the whole-body net balance (anabolic) response was correlated with the EAA content of the protein food source (*p* < 0.001) (Figure 1). These data illustrate the limitations of dietary guidelines failing to consider protein quality.

### Beyond protein quality: the significance of non-protein components of protein food sources

DIAAS quantifies the quality of a single protein or a group of proteins. Neither DIAAS nor any other measure of protein quality accounts for the non-protein components of a dietary protein food source. However, apart from nutritional supplements, dietary protein is normally consumed in a food source that contains non-protein components. Dietary carbohydrate and fat components of protein food sources may potentially affect many aspects of the physiological response, including the net gain in body protein (the anabolic response). Carbohydrate is well known to amplify the protein synthetic response to dietary protein (89). Dietary fat also increases the magnitude of response to dietary protein. For example, whole milk increases the protein synthetic response to dietary protein as compared to the same amount of protein in the form of skim milk (90). Furthermore, an acute increase in plasma fatty acids improved muscle protein synthesis despite inducing insulin resistance (91).

**Figure 2:**
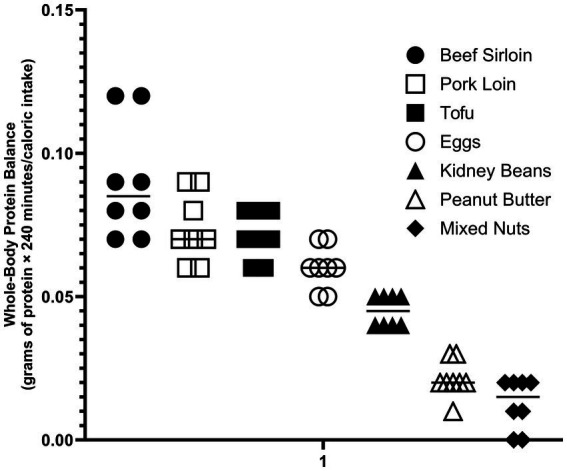
Anabolic response determined by stable isotope tracer methodology of ounce equivalents protein food sources normalized for energy content of the non-protein components (81).

It is not obvious how the role of the non-protein components of protein food sources can be included in the assessment of protein quality. One approach is to normalize the anabolic response to the protein food source by the corresponding caloric value. In the example of the ounce equivalent protein food sources discussed above, the protein food sources with the highest DIAASs (beef, pork, eggs and tofu) stimulated the anabolic response with less caloric intake than those with the lower DIAASs (kidney beans, peanut butter, and mixed nuts) (Figure 2).

**Figure 3:**
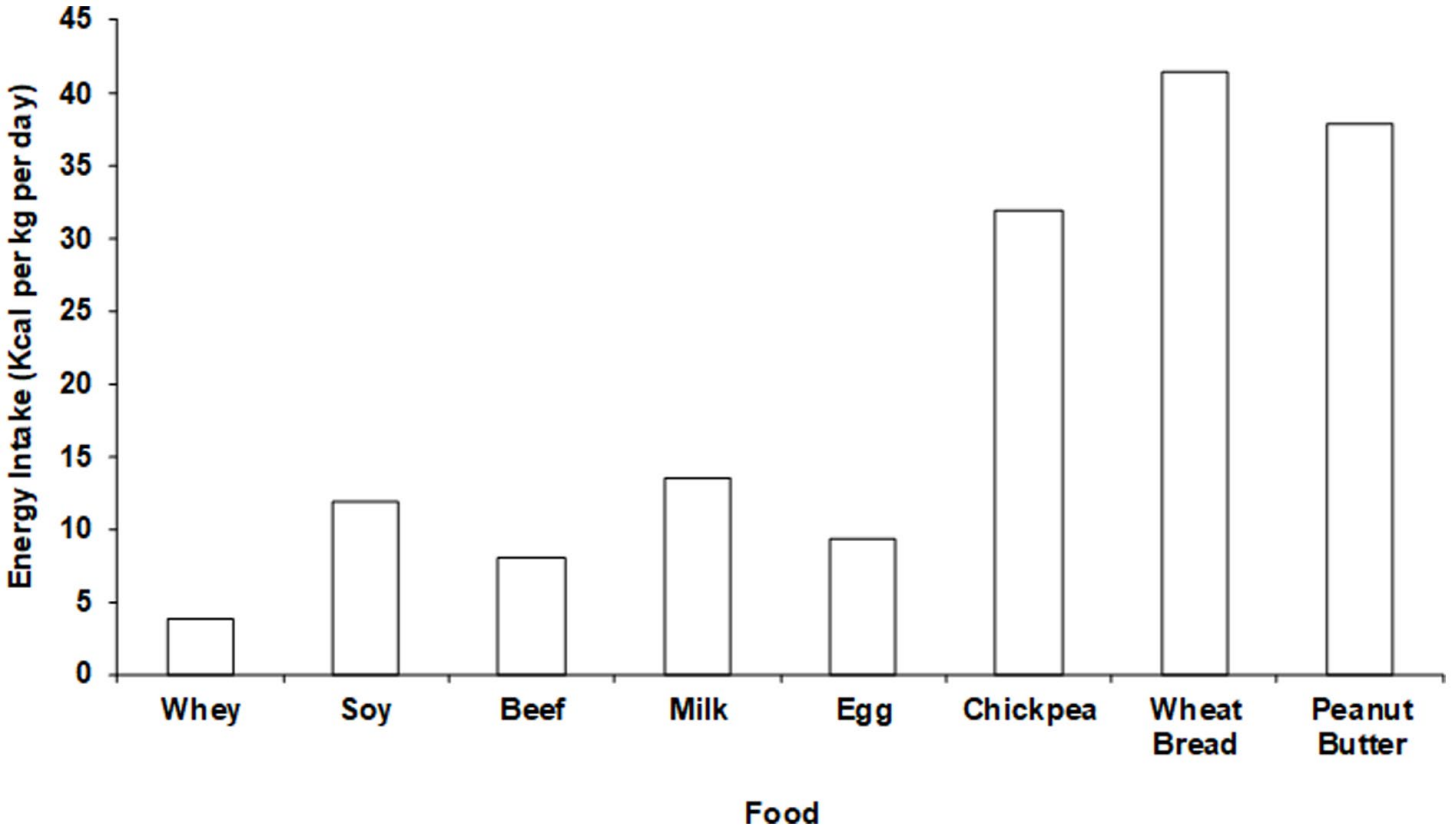
Net whole-body protein balance per calorie of intake with different “ounce equivalent” protein food sources.

The potential physiological significance of the non-protein components of protein food sources can be appreciated by calculating the caloric content of the amount of each dietary protein food source that would be required to fully meet all EAA requirements (Figure 3). For lower quality proteins such as nuts and beans the entire diet would have to be comprised of only those protein food sources to avoid a positive energy balance (i.e., weight gain). These data highlight the importance of considering the total caloric content when translating DIAASs to dietary recommendations. Accounting for the non-protein components of protein food sources is particularly important during caloric restriction weight loss (CRWL). CRWL induces a negative energy balance that impairs the anabolic response to dietary protein. For that reason, an intake of at least 1.2 g protein /kg/day is necessary to maintain muscle mass during CRWL (92). To reach this goal, it is necessary to rely entirely on high quality protein food sources so that the accompanying caloric value associated with the non-protein components is minimized.

### Protein quantity vs. quality

Can consumption of more of the same dietary protein food sources compensate for low protein quality? This question can be addressed by considering the *utilizable protein* in a diet pattern. The utilizable protein in a single mixed meal or the entire daily protein consumption can be calculated by multiplying the overall DIAAS (as described in ref. 13) by the amount of protein consumed. The rationale underlying this approach is that the synthesis of complete proteins from dietary EAAs requires the availability of all the EAAs, and when the demand for the limiting EAA exceeds the amount of that EAA absorbed, further protein synthesis from the non-limiting dietary EAAs cannot proceed.

To illustrate the interaction of dietary protein quantity and quality we will consider a simplified numerical example in which the daily intake of dietary protein is comprised of two proteins, with one of the proteins being low-quality protein (DIAAS = 50%) and one being high-quality (DIAAS = 100%), and to simplify the math the limiting amino acid is assumed to be the same for both proteins. We will assume that total daily protein intake is 50 g, which would correspond to a 70 kg person consuming slightly more than the EAR (0.66 g protein/kg/day), and that 25 g of each protein is consumed. The DIAAS of this combination would be 75%, and the utilizable protein is (25 g × 0.5) + (25 g × 1.0) = 37.5 g, which would be less than the EAR. If consumption of the low-quality protein is increased to 50 g and the high-quality protein consumption remains at 25 g, the overall DIAAS would be reduced to 66%, but the total utilizable protein consumption would increase to 49.5 g (approximately equal to the EAR) because of the increase in total protein consumption. However, this approach would require consumption of 75 g of dietary protein, which may be difficult for some to achieve due to issues of cost, taste and convenience. These factors are important drivers of food consumption (93). Further, since low-quality proteins are usually plant based (14), increasing consumption would likely significantly increase the associated caloric content of the protein food sources of the diet. If we consider another example in which the initial parameters are the same, but the consumption of the high-quality protein is increased to 50 g while consumption of the low-quality protein is maintained at 25 g, the overall DIAAS would increase to 83%. The product of DIAAS and protein consumption would increase the utilizable protein to 62.5 g and would likely involve a smaller increase in caloric intake than when the low-quality protein consumption is increased. Thus, increasing the quantity of both low-quality and high-quality protein can help to meet dietary EAA targets, but the increase in EAA consumption will be greater when the overall DIAAS is increased by increasing the consumption of the high-quality protein.

This simplified example illustrates that, when the limiting amino acid is the same for different dietary proteins, increasing the amount of high-quality protein consumed increases the utilizable protein more effectively than increasing the amount of low-quality protein consumed. However, in more realistic dietary patterns, proteins may be complementary, due to the limiting amino acids being different. The amount that complementary proteins improve the overall quality of the dietary protein (i.e., DIAAS) is dependent on the magnitude of the difference between the EAA content relative to the reference protein of the limiting EAAs of the two proteins. In addition, the EAA content relative to the reference protein for next-limiting EAAs in the two proteins will impact the extent to which the proteins are complementary. While specific numerical examples would be complicated, some generalizations are possible. Complementary low-quality proteins have the potential to increase the DIAAS more than with complementary high-quality proteins, because the discrepancies between the limiting EAAs are likely to be greater with low-quality proteins. High-quality proteins have DIAASs >100%, meaning that there is not a large difference between the values for the limiting EAAs (14). While combining complementary low-quality dietary proteins will increase the utilizable protein, this approach will not achieve the same increase in utilizable protein as combining a low-quality protein with a high-quality protein, particularly if the limiting EAAs in the low- and high-quality proteins differ (i.e., they are complementary). Combining low- and high-quality complementary proteins will result in a DIAAS greater than the DIAAS of the low-quality protein, but lower than the DIAAS of the high-quality protein, with corresponding impact on the amount of utilizable protein.

### Differences in the metabolic fate of EAAs

Nutritional guidelines for dietary protein have been derived from whole-body measurements, primarily N-balance or isotopic tracer methods. However, differences in tissue- and organ-specific responses may arise in response to varied protein food sources that are not evident from the whole-body responses yet have physiological significance. For example, the response of peripheral blood levels of EAAs following consumption of soy protein is limited by extensive splanchnic clearance of absorbed EAAs (94, 95). As a result, there may be minimal stimulation of muscle protein FSR by soy protein consumption (96), even though the whole-body protein net balance response (which includes splanchnic uptake) is comparable to that following consumption of the same amount a different high-quality protein such as beef. Consumption of beef protein, on the other hand, results in a relatively rapid and greater total plasma EAA response than soy, with a corresponding greater stimulation of MPS (92). The differences in splanchnic and peripheral responses to dietary protein are demonstrated by the results of a recent study we performed comparing the MPS and whole- body protein responses to consumption of a 4 oz. beef patty vs. the responses to consumption of a 4 and to an 8 oz (97). “Impossible Burger” comprised of soy-based protein. The response of plasma EAA concentrations was greater following consumption of the 4 oz. beef patty than the 4 oz. Impossible Burger, which corresponded to the differences in EAA contents of the two proteins. As a result, both whole-body and MPS were significantly stimulated by the beef patty, but neither were stimulated by consumption of the 4 oz. Impossible burger (98). More relevant to the issue of differing fates of ingested EAAs, the response of plasma EAAs following consumption of 4 oz. beef burger was greater than the Impossible Burger despite the greater total EAA content of the 8 oz. Impossible Burger (corrected for the lower digestibility of soy protein) (Figure 3). As a result of greater splanchnic extraction of absorbed EAAs following soy protein consumption, MPS was stimulated to a greater extent by the 4 oz. beef patty than the 8 oz. of Impossible Burger despite equivalent increases in whole body net protein balance (98).

### Is protein quality relevant in high income countries?

**Figure 4:**
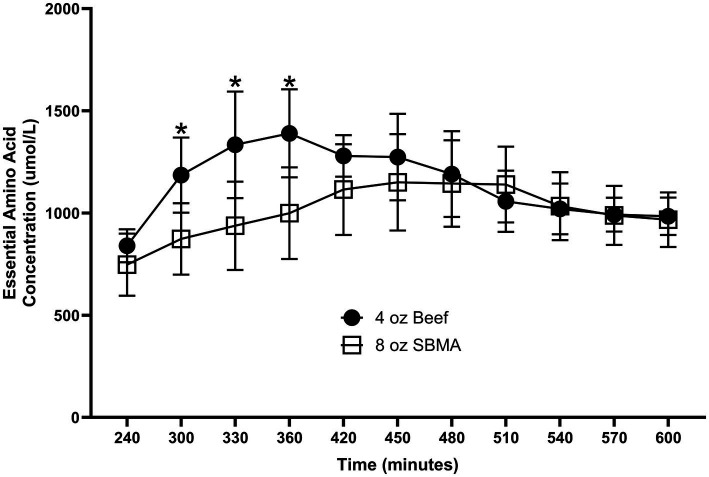
Energy requirements to meet minimal EAA requirements with different “ounce equivalent” protein food sources. Total energy requirements are approximately 35 kcal/kg/day (Adapted from (98), licensed under CC BY 4.0).

Average protein consumption in underdeveloped countries may be insufficient to meet all EAA requirements (99). In high-income countries there is less concern that dietary protein consumption is inadequate to provide adequate EAAs. Rather, the notion that dietary protein is “over-consumed” is commonly expressed in publications ranging from scientific to lay articles in high-income countries. However, a careful analysis of dietary protein intake in high income countries that takes protein quality into account has been lacking. This issue has recently been addressed by analyzing the implications of variations in dietary protein quality for the adequacy of dietary protein intake in the United States (100). The analysis used published FAO food supply data sets giving overall total protein intakes, as well as NHANES survey data across a well described population. Account was taken of potential differences in dietary protein quality, as quantified by DIAAS. Data were analyzed for healthy adults, as well as for specific nutritional states that may affect the optimal level of protein and EAA consumption, such as caloric restriction weight loss diets, aging, aerobic and resistance exercise training, and vegan/vegetarian diets. Protein consumption data were compared with both the EAR (0.66 g protein/kg/day) and the RDA (0.83 g protein/kg/day) for different populations. To account for protein quality, the utilizable protein intake (calculated as described above) was calculated (Figure 4).

Data from the US National Health and Nutrition Examination Surveys for 2001–2018 was used to assess the percentage of the adult population having utilizable protein intakes potentially less than recommended levels. Utilizable protein intake was calculated for DIAASs ranging from 1.0 to 0.6. An analytical sample of 44,018 (22,079 males and 21, 939 females) was used, stratified by age and gender. 11% of the adult population had estimated utilizable protein intakes below the EAR even if a DIAAS of 1.0 is assumed (i.e., all protein consumption was “high quality” protein), and the percentage increased to 20% in the 71+ age group if the DIAAS of the total protein intake was 1.0. The percentage of the population 19–50 year of age consuming protein intakes below the EAR when DIAAS was assumed to be 1.0 was higher for women than men (16% versus 5%), and the percentage increased with age (71+ years male and female = 20%). The percentage of the population with utilizable protein intakes potentially falling below the EAR increased considerably as DIAAS declined, with potentially 72% of the 71+ year-old population having utilizable protein intakes falling below the EAR if DIAAS was taken to be 0.6, and that number increased to 88% if compared to the RDA. While these values are theoretical, the data analysis highlights the potential importance of protein quality in meeting EAA requirements, even in a high-income country.

## Conclusion

Dietary protein quality, defined generally as the ability to provide to the body an optimal amount and profile of EAAs per gram protein consumed, in accord with dietary requirements, varies between proteins. EAAs cannot be synthesized in the body, and consumption of at least the RDAs for each EAA is required for optimal protein nutrition. Currently, protein quality can most accurately be quantified by the DIAAS, although DIAAS has potential shortcomings when applied to dietary planning. DIAAS is based on the EAR for protein, and individual dietary planning will most commonly be based on the RDA for protein.

When account is taken of protein quality by means of the DIAAS, utilizable dietary protein may fall below the amount needed to meet EAA requirements, even in high-income countries. Moreover, optimal EAA consumption is likely well above the minimal acceptable amount in a wide range of metabolic and physiological circumstances. In such circumstances it is important that dietary protein consumption is composed largely of high-quality proteins. Reliance on low quality proteins to meet elevated EAA recommendations will usually involve a significant increase in caloric intake due to the non-protein components of the low-quality protein food source. The next major advance in protein/amino acid nutrition will be the tailoring of dietary patterns to individual needs, predicated on the metabolic and physiological state of the individual. This progression will require better understanding of optimal levels of EAA consumption in different circumstances, coupled with use of a scoring system such as DIAAS, to quantify the utilizable protein in a dietary pattern.
